# Supplementary material for: Fusion Protein of RBP and Albumin Domain III Reduces Lung Fibrosis by Inactivating Lung Stellate Cells
Source: Biomedicines. 2023 Jul 16;11(7):2007. doi: 10.3390/biomedicines11072007 (PMC10377390; doi:10.3390/biomedicines11072007)
Supplement: Supplementary file 1 [file biomedicines-11-02007-s001.zip › biomedicines-2493355-supplementary.pdf]

Supplementary Materials

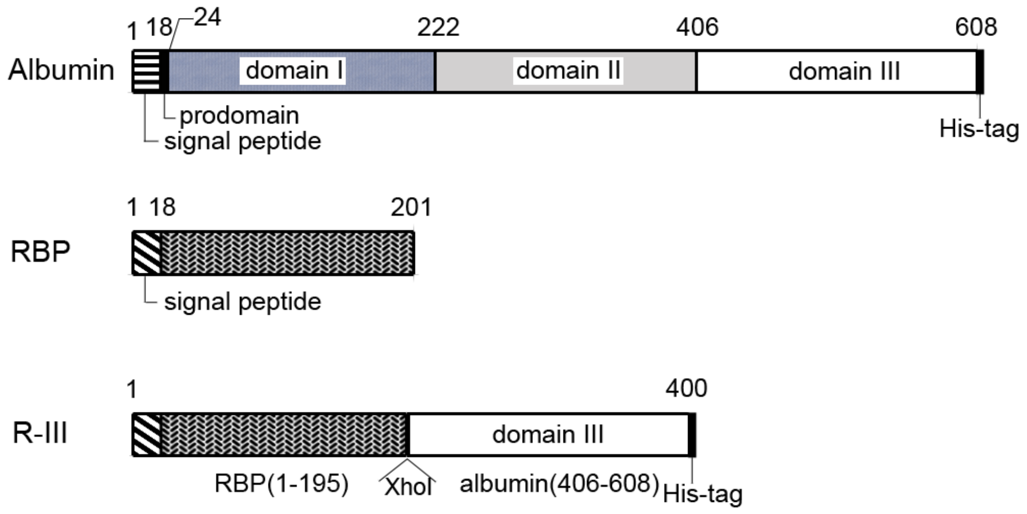

Figure S1. Schematic diagram of the retinol-binding protein (RBP)–albumin domain III fusion protein, in comparison with full-length albumin and RBP. Note that the fusion protein is histidine tagged at the C-terminal end. The numbers indicate amino acids.

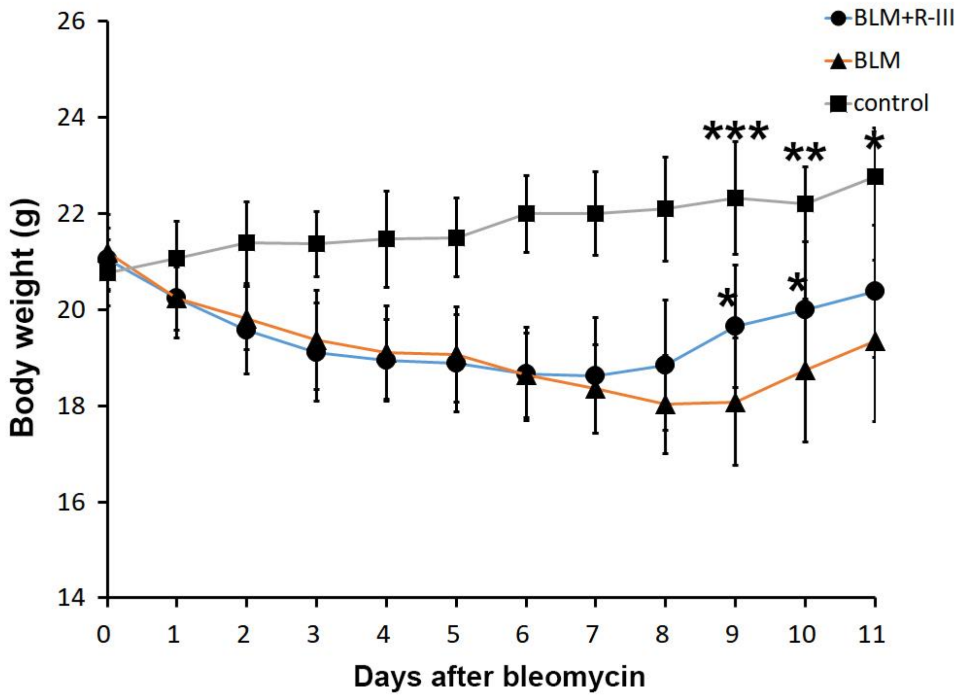

Figure S2. Body weight of control, bleomycin (BLM)-, BLM+R-III-treated mice. The body weight of the mice was monitored over a period of 11 days after bleomycin instillation and expressed as means ± SD. * $p < 0.05$, ** $p < 0.01$, and *** $p < 0.001$ by a Welch's t-test (compared with BLM-treated mice).

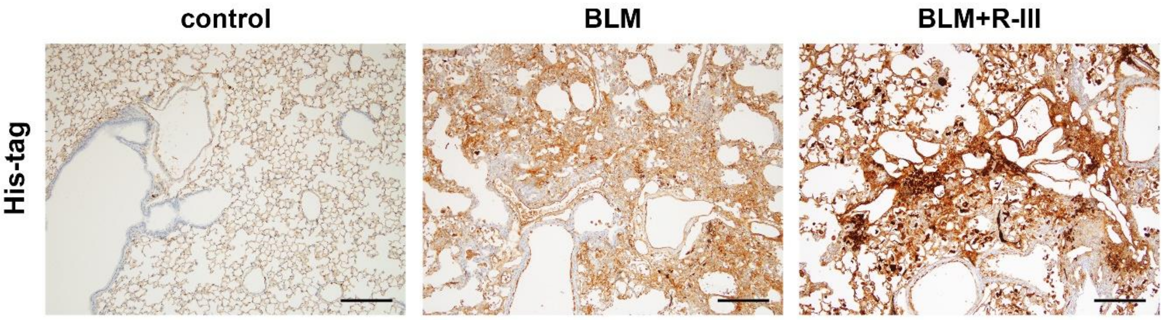

Figure S3. Cellular distribution of injected His-tagged R-III. Lung sections from control, BLM-treated, and BLM+R-III-treated mice were subjected to immunohistochemical staining for the His-tag. The His-positive signals were observed to be localized in the fibrotic foci. Scale bar, 200 μm.
